# Spiritual Writings

**Published:** 1852-05

**Authors:** 


### THE WESTERN JOURNAL.

THIRD SERIES.—VOL. IX.—NO. V.

LOUISVILLE, MAY 1, 1852.

SPIRITUAL WRITINGS.

The extracts under this head in our last number occupied more space than we supposed they would, and as a consequence we were not able to conclude what we wished to say in regard to the *Spiritual Writings;* but our readers need not be alarmed—we shall not weary their patience much longer with our dissertation on this subject.

We have said that there appears to us to be a striking analogy between the condition of the nervous system which leads to these writings, and that which existed in the persons who were affected with the "jerks;" and some further facts which we have now to add will, we think, render this still more apparent. Thus, while this singular affection was not confined to any class or sex, but men and women, black and white, were its subjects, still it was observed that women were much more apt to fall into it than men; and it was also remarked that those who had once been seized were particularly liable to a second attack, and jerking or swooning readily became a habit. "Women," it is stated, "had their nerves so weakened by the frequency of these attacks, as to fall while walking to or from the meeting-house, engaged in narrating past exercises without any uncommon emotion, and drop from their horses on the road."

Many instances of this acquired habit of the nervous system are recorded by the writers of that period. Thus, Dr. Cleland, an estimable and pious clergyman, relates that riding one day with a lady, the wife of a presbyterian elder, who had been sometime previously affected with the jerks, it occurred to him to try whether they might not be renewed simply by starting a particular train of ideas in her mind. The conversation just before had been of an indifferent character; he changed it abruptly to devout and solemn subjects, and adds, that "before two minutes had elapsed, her body began to be violently agitated, pitching upward and forward, from the saddle halfway to the horse's neck six or eight times in a minute."

There were those who struggled long and earnestly against the disposition to fall, but were forced to yield at last. One fell, after bitterly opposing what was esteemed a "divine work," and another, exclaiming that it was "an unfortunate sight and a great mortification." "One dropped, as if shot, just after expressing his fears that the work was not right." A father threatened his swooning daughters that he would beat them if they ever came to such a place again, and fell with the words in his mouth. A man fell at Lexington, "who had told an acquaintance if he fell he might put his foot on his neck and keep him down."*

Not only were there these involuntary motions, the result of sympathy, but in many of the subjects there was also the unconsciousness and insensibility presented by the mesmeric state. Persons, to their great surprise, found themselves unable to move when they wished. One young lady is mentioned who was not aware of any change in her condition, and was amazed to find the people flocking around her; but then making an effort to move she found herself powerless. Some, while in this state, were both conscious and capable of conversing; others were speechless. The most energetic stimulants, as in artificial somnambulism, made no impression upon the sentient nerves. A phial of harts-

*Davidson's Hist. Presb. Church in Kentucky.

horn was applied by a clergyman to the nose of a stout young man, who was lying flat on his back, and by accident some got into his nostrils; "but he took not the slightest notice of it."

On one occasion Lorenzo Dow, while preaching in the court-house at Knoxville, Tenn., the Governor of the State being present, saw one hundred and fifty persons exercised with the jerks. At another meeting, where the excitement had risen to a wilder pitch, three thousand persons were reported to have fallen. The influence by which these strange manifestations were induced, as every one must be prepared to learn, was held by the multitude to be supernatural. It was esteemed, as we have said, a divine work, which it was hazardous and sinful to oppose. The subjects were often in an ecstatic state, and had visions and revelations. They saw dazzling light such as they could not behold. "Two women," says a historian of the times, "have fallen into trances, and one has passed a golden bridge to heaven; the other has been in heaven," &c., &c.

No doubt there were sensible and discreet men, probably physicians, who believed that these people were in communication with the spiritual world. No one believes so now; and yet the "spiritual writers" may be defied to bring out anything more marvelous than the phenomena afforded by the "jerks." These things—mesmerism, the jerks, spiritula writing, all in them that is not fraud and deception—belong, then, in our judgment, to the same category, and have their origin in a peculiar, perverted state of the brain. It is a state easily induced in some individuals, while others are capable of resisting it. The subjects of mesmerism are found to be the apt spiritual writers, as the believers in clairvoyance are those who yield the readiest credence to its being the work of spirits.

We do not deem it worth while to write against the thing. It *will* have its day. The populace will be carried away with it; some will lose their senses, and commit crimes, or get into mad-houses; and then, after a time,

> "——— ——— all this derision
> Will seem a dream, and fruitless vision."

As an apology for the length to which we have extended our remarks upon this subject we have said, that the miserable superstition has ended, in not a few instances, in deplorable insanity. In this city we already hear of persons who are fully persuaded that they hold daily converse with the spirits of their departed friends, and one young man is understood to have been impelled to suicide by these spiritual writers. We may not be able to arrest the delusion, but it ought, at least to be exposed.

